# Correlation analysis of systemic immune inflammatory index, Serum IL-35 and HMGB-1 with the severity and prognosis of sepsis

**DOI:** 10.12669/pjms.39.2.6651

**Published:** 2023

**Authors:** Ke Ma, Yajuan Zhang, Jian Hao, Jing Zhao, Ying Qi, Cong Liu

**Affiliations:** 1 Ke Ma, Clinical Laboratory, The First Affiliated Hospital of Hebei North University, Zhangjiakou 075000, Hebei, China; 2 Yajuan Zhang, Clinical Laboratory, The First Affiliated Hospital of Hebei North University, Zhangjiakou 075000, Hebei, China; 3 Jian Haom, Clinical Laboratory, The First Affiliated Hospital of Hebei North University, Zhangjiakou 075000, Hebei, China; 4 Jing Zhao, Clinical Laboratory, The First Affiliated Hospital of Hebei North University, Zhangjiakou 075000, Hebei, China; 5 Ying Qi, Clinical Laboratory, The First Affiliated Hospital of Hebei North University, Zhangjiakou 075000, Hebei, China; 6 Cong Liu, Clinical Laboratory, Ganzhou City People’s Hospital, Ganzhou 341000, Jiangxi, China

**Keywords:** Systemic Immune Inflammatory Index, Interleukin-35, High Mobility Group-Box one, Sepsis, Prognosis

## Abstract

**Objective:**

To investigate the correlation between systemic immune inflammatory (SII) index, serum interleukin-35 (IL-35) and high mobility Group-Box one (HMGB-1) with the severity and prognosis of sepsis.

**Methods:**

A retrospective analysis was performed on the clinical data of 209 patients with sepsis admitted to Ganzhou City People’s Hospital from October 2019 to October 2021. One hundred eighteen patients in Group-A had common sepsis, and 91 patients in Group-B had septic shock, which were subdivided into the survival Group-And the mortality Group-According to the 28d prognosis. The levels of SII, IL-35 and HMGB-1 in different groups were compared, and their correlation with the severity and prognosis of sepsis was analyzed.

**Result:**

The levels of SII, IL-35 and HMGB-1 in Group-A were significantly lower than those in Group-B (p<0.05). The levels of SII, IL-35 and HMGB-1 in the survival group were significantly lower than those in the death group (p<0.05). Spearman correlation analysis showed that the levels of SII, IL-35, and HMGB-1 were significantly positively correlated with the severity of sepsis (p<0.05), and significantly positively correlated with the prognosis of patients with sepsis (p<0.05).

**Conclusion:**

SII, IL-35 and HMGB-1 are remarkably correlated with the severity and prognosis of patients with sepsis. With the increasing in SII, IL-35 and HMGB-1 levels, patients will suffer from severe disease progression and poor prognosis, which require more clinical attention.

## INTRODUCTION

Sepsis, which is caused by infection of the body, is one of the diseases with high incidence and mortality in recent years. It usually manifests as an excessive inflammatory response to form a systemic inflammatory response syndrome, which causes damage to a variety of cells and tissues in the human body.^1^ Sepsis progresses rapidly. If it cannot be effectively intervened and controlled, multiple organ failures and a series of serious complications will be further induced, resulting in septic shock and other adverse outcomes.^2^ Consequently, how to efficiently and accurately predict the disease progression and prognosis of patients is the key to reducing the occurrence of adverse events.^3^

Nowadays, clinical indexes such as interleukin-6 (IL-6), acute physiology and chronic health status scoring system are commonly used to evaluate the disease profile of patients,^4^ but all of them show unsatisfactory clinical value. But their clinical value is not ideal. Therefore, it is of great importance to seek novel and efficient indexes for early diagnosis, treatment and prediction. Previous studies have shown that the disease evolution process of sepsis is relatively complicated, and abnormal immune function is the key to the severe disease of patients. Meanwhile, a large number of inflammatory factors are also involved in the whole process.^5^ SII, as a scoring system for evaluating the immune function of tumor patients, can effectively predict the prognosis of patients.^6^ IL-35 is a new member of interleukin and an important anti-inflammatory cell, which has a close bearing on the immune level of patients.

Experiments on mice showed that the expression level of interleukin in mice with septic shock and sepsis was significantly increased.^7^ HMGB-1 is an inflammatory mediator with a variety of biological effects such as mediating inflammatory response and regulating gene transcription, which plays a vital role in the occurrence and progression of sepsis.^8^ Currently, there are still studies exploring the role of IL-35 levels in patients with sepsis, but very few studies on the correlation between SII and HMGB-1 levels and sepsis have been carried out. Based on this, in this study, the clinical data of 209 patients were analyzed and compared, aiming to investigate the correlation of the above indexes with the severity of the disease and prognosis of the patients, so as to provide a basis for the ideal quality of prognosis of these patients.

## METHODS

A total of 209 patients with sepsis admitted to Ganzhou City People’s Hospital from October 2019 to October 2021 were selected as subjects, and their clinical data were retrospectively analyzed. All 209 patients were divided into Group-A and Group-B according to the severity of their disease. Among the 118 patients in Group-A, there were 67 males and 51 females, ranging in age from 33 to 74 years old, with an average age of (55.65 ± 8.14) years. Among the 91 patients in Group-B, there were 54 males and 37 females, ranging in age from 35 to 71 years old, with an average age of (56.01 ± 7.97) years. No statistically significant difference was observed in the general clinical data of the two groups (p>0.05), which were comparable, see (Table-I). The patients were subdivided into the survival group (167 cases) and the death group (42 cases) according to the 28 day prognosis and survival. The study was approved by the Institutional Ethics Committee of The First Affiliated Hospital of Hebei North University on February 2022 No. (2022-16).

**Table-I:** Comparison of general data between Group-A and Group-B n (%), (*χ̅*±*s*).

Group	Number of cases	Gender		Age (years old)	BMI (kg/m^2^)	History of hypertension	History of diabetes	History of coronary heart disease	Heart rate (beats/min)	Systolic blood pressure	Diastolic pressure (mmHg)	Primary disease		
	
		Male	Female									Lung infection	Intraperitoneal infection	Other
Group-A	118	67 (56.78)	51 (43.22)	55.65±8.14	23.54±2.41	35 (29.66)	23 (19.49)	18 (15.25)	83.00±9.65	130.65±25.65	80.56±8.41	70 (59.32)	24 (20.34)	24 (20.34)
Group-B	91	54 (59.34)	37 (40.66)	56.01±7.97	23.75±2.38	27 (29.67)	17 (18.68)	12 (13.19)	82.76±9.42	131.16±24.98	81.30±7.99	55 (60.44)	19 (20.88)	17 (18.68)
χ^2^/Z/t		0.138		0.320	0.628	0.000	0.022	0.179	0.180	0.144	0.644	0.221		
P		0.710		0.749	0.531	0.999	0.883	0.673	0.857	0.886	0.520	0.825		

### Inclusion criteria:

- Patients who meet the diagnostic criteria for sepsis in the Guidelines for the Treatment of Severe Sepsis/Septic Shock in China (2018)^9^
- Patients with complete clinical data;
- Patients who are informed and meet the ethical review criteria set out in the Helsinki Declaration;
- Patients who have not received relevant treatment recently;
- Patients without cognitive impairment;
- Women without pregnancy and breastfeeding;
- Patients without other malignant tumors.

### Exclusion criteria:

- Patients complicated with hematological diseases;
- Patients who died within a short period of hospitalization;
- Patients with acute or chronic serious diseases;
- Patients with autoimmune dysfunction;
- Patients with organ failure;
- Patients with mental diseases.

Platelet count, lymphocyte count and neutrophil count were detected by Countess II FL automatic cell analyzer produced by Thermo Fisher Scientific, SII=platelet count × neutrophil count/lymphocyte count. Five ml of fasting elbow venous blood was collected from all patients on the 1st day of admission, centrifuged at 3500 r/min for 15 minutes, and then the upper serum was separated. Subsequently, the blood was stored at -40°C for testing, and the levels of IL-35 and HMGB-1 were detected by enzyme linked immunosorbent assay (ELISA). The instrument was Mind ray BS-2000M automatic biochemical analyzer, and the kit was purchased from Shanghai Huzheng Biotechnology Co., Ltd.

### Statistical analysis:

All data in this study were analyzed by SPSS22.0 statistical software. Count data were expressed by N (%), χ^2^ test was performed for count data, which was expressed by n (%), and rank sum test was used for rank data; All measurement data were tested by t-test and represented by (); Spearman method was used for correlation analysis, and p<0.05 indicated a statistically significant difference.

## RESULTS

The levels of SII, IL-35 and HMGB-1 in Group-A were significantly lower than those in Group-B, with a statistically significant difference (p<0.05). Table-II Spearman correlation analysis showed that the levels of SII, IL-35, and HMGB-1 were significantly positively correlated with the severity of sepsis, and the data results were all statistically significant (p<0.05. Table-III The levels of SII, IL-35 and HMGB-1 in the survival group were significantly lower than those in the death group, with a statistically significant difference (p<0.05). Table-IV. Spearman correlation analysis showed that the levels of SII, IL-35 and HMGB-1 were significantly positively correlated with the prognosis of patients with sepsis, and the data results were all statistically significant (p<0.05). Table-V

**Table-II:** Comparison of SII, IL-35 and HMGB-1 levels in groups A and B (*χ̅*±*s*).

Group	Number of cases	SII	IL-35 (ng/L)	HMGB-1 (μg/L)
Group-A	118	392.65±51.39	48.61±6.47	119.61±21.56
Group-B	91	1454.32±268.08	56.44±7.21	163.50±19.72
t		42.058	8.252	15.139
P		0.000	0.000	0.000

**Table-III:** Correlation analysis between the levels of SII, IL-35 and HMGB-1 and the severity of sepsis.

		Severity of illness	SII	IL-35	HMGB-1
Severity of illness	Correlation coefficient	1	0.859**	0.514**	0.735**
	Significance (two-tailed)	.	0.000	0.000	0.000
	N	209	209	209	209
SII	Correlation coefficient	0.859**	1	0.631**	0.793**
	Significance (two-tailed)	0.000	.	0.000	0.000
	N	209	209	209	209
IL-35	Correlation coefficient	0.514**	0.631**	1	0.676**
	Significance (two-tailed)	0.000	0.000	.	0.000
	N	209	209	209	209
HMGB-1	Correlation coefficient	0.735**	0.793**	0.676**	1
	Significance (two-tailed)	0.000	0.000	0.000	.
	N	209	209	209	209

** Correlation was significant at the 0.01 level (two-tailed).

**Table-IV:** Comparison of SII, IL-35 and HMGB-1 levels between the survival Group-And the death group (*χ̅*±*S*).

Group	Number of cases	SII	IL-35 (ng/L)	HMGB-1 (μg/L)
Survival group	167	270.90±46.32	37.20±6.67	107.36±22.30
Death group	42	2106.46±233.62	80.36±15.59	175.90±28.84
t		94.993	27.310	16.726
P		0.000	0.000	0.000

**Table-V:** Correlation analysis between the levels of SII, IL-35, HMGB-1 and the prognosis of patients with sepsis.

		Prognosis	SII	IL-35	HMGB-1
Prognosis	Correlation coefficient	1	0.694**	0.687**	0.644**
	Significance (two-tailed)	.	0.000	0.000	0.000
	N	209	209	209	209
SII	Correlation coefficient	0.694**	1	0.694**	0.612**
	Significance (two-tailed)	0.000	.	0.000	0.000
	N	209	209	209	209
IL-35	Correlation coefficient	0.687**	0.694**	1	0.771**
	Significance (two-tailed)	0.000	0.000	.	0.000
	N	209	209	209	209
HMGB-1	Correlation coefficient	0.644**	0.612**	0.771**	1
	Significance (two-tailed)	0.000	0.000	0.000	.
	N	209	209	209	209

** Correlation was significant at the 0.01 level (two-tailed).

## DISCUSSION

Systemic inflammatory network efficiency is one of the important pathogenesis of sepsis. Patients with sepsis show insignificant clinical symptoms at the early stage, and still have a poor prognosis even with corresponding treatment measures after symptoms become apparent.^8^,^9^ In view of the high incidence and mortality of sepsis, clinical attention should be paid to the exploration of indexes for disease progression and prognosis prediction. Early analysis and prediction of a patient’s disease may provide a plan for early diagnosis as well as treatment and prevention.^10^,^11^

The results of this study showed that the levels of SII, IL-35 and HMGB-1 in Group-A were significantly lower than those in Group-B, and those in the survival group were all lower than those in Group-B. Further correlation analysis showed that the three indexes of SII, IL-35 and HMGB-1 were significantly positively correlated with the disease severity and prognosis of patients with sepsis, suggesting that patients with sepsis showed an increased inflammatory response and decreased immune function, and the inflammatory status and immune level were unbalanced with the development of the disease.

This could be caused by: (1) SII is a predictor reflecting the quality of life and recurrence of patients with tumors. It plays a preferable role in evaluating whether immune imbalance occurs in the body and is an efficient and accurate scoring system.^12^,^13^ SII stands for platelet count multiplied by neutrophil count/lymphocyte count. Patients with immune system disorders are mostly caused by inflammatory responses, resulting in a significant increase in the levels of platelet, neutrophil and lymphocyte counts. Both neutrophil and lymphocyte counts are cytokines reflecting immune function.

When patients suffer from sepsis, the two kinds of cells will be separated, in which the expression of neutrophils increases, while that of lymphocytes decreases, which is manifested as apoptosis of T cells.^14^ From this point of view, SII can be effectively applied in the assessment of septic disease progression and prognosis. IL-35 is an immunosuppressive cytokine generated by T cells, which plays a role in maintaining the immune tolerance of the body. Previous studies have confirmed that the expression level of IL-35 increases significantly in many autoimmune diseases.^15^,^16^ Sepsis is also a systemic inflammatory disease. With the progression of the disease, the expression level of IL-35 increases significantly.

In such a case, patients may be in a state of immune paralysis, where anti-inflammatory mediators dominate. As a result, they need to secrete a large amount of IL-35 to produce a protective effect,^17^ resulting in an abnormal increase in the expression level of this index. HMGB-1 is a chromosome-binding protein with multiple functions such as stabilizing nuclear structure and mediating the inflammatory response. It has a pro-inflammatory effect in the body of patients with sepsis, possibly because it promotes the generation of inflammatory factors such as IL-35 and IL-6 by activating neutrophils and mononuclear macrophages, thus participating in the process of the systemic inflammatory response.^18^ Furthermore, HMGB-1 has a damaging effect on vascular endothelial, leading to a decrease in vascular endothelial function and organ function injury in patients.^19^,^20^ The conclusions of this study enriched the clinical data on the correlation between the levels of SII, IL-35 and HMGB-1 and the severity and prognosis of sepsis, and further verified the clinical significance of monitoring these indicators in clinic.

### Limitations:

It includes a retrospective analysis, and there are problems of insufficient design and foresight. In view of this, further improvements will be made in future research to make more scientific research results.

## CONCLUSION

Patients with sepsis show a remarkable increase in the three levels of SII, IL-35 and HMGB-1, which are significantly positively correlated with the disease severity and prognosis of patients. The higher the expression levels of the three indexes, the more severe the progression of patients and the poorer the prognosis. To this end, the level of the three indexes needs to be monitored clinically.

### Authors’ Contributions:

**KM and CL** designed this study and prepared this manuscript, and are responsible and accountable for the accuracy or integrity of the work.

**YZ and JH** collected and analyzed clinical data.

**JZ and YQ** significantly revised this manuscript.
